# Student nurses’ practices and willingness to teach relatives breast self-examination in Nigeria

**DOI:** 10.4102/hsag.v29i0.2494

**Published:** 2024-01-25

**Authors:** Joel O. Aluko, Olayinka A. Onasoga, Regis R. Marie Modeste, Odinaka B. Ani

**Affiliations:** 1 Department of Nursing Sciences, College of Health Sciences, University of Ilorin, Ilorin, Nigeria; 2 Department of Nursing and Midwifery, Faculty of Medicine and Health Sciences, Stellenbosch University, Tygerberg, South Africa; 3 Department of Nursing, Faculty of Health, Sports and Bioscience, University of East London, East London, United Kingdom

**Keywords:** nursing students, practice, teaching, breast self-examination, relatives, Nigeria

## Abstract

**Background:**

Breast cancer is the most common cancer and the leading cause of cancer-related death for women worldwide. Breast self-examination (BSE) is an essential, low-cost, and simple tool for detecting breast cancer early. Employing the idea of ‘charity begins at home’ by involving student nurses in teaching BSE to relatives will improve early detection.

**Aim:**

To assess nursing students’ practice and willingness to teach BSE to their relatives.

**Setting:**

A college of nursing and midwifery in one state under North-Central Nigeria.

**Methods:**

A cross-sectional descriptive design was employed. Through incidental sampling technique 197 respondents were selected from the first to the third year. Data were collected using a structured questionnaire. Descriptive and inferential analyses, with a *p*-value of 0.05 were conducted.

**Results:**

Respondents indicated where they learned about BSE. There were 98.5% respondents who had heard about BSE, and 89.8% of them had good practice of BSE. However, a quarter did not teach BSE to relatives. There were no statistically significant associations noted.

**Conclusion:**

Most of the nursing students were aware of BSE and knew how to perform it, although a quarter did not teach BSE to their relatives. Therefore, it may be necessary to sensitise nurses to cultivate the habit of teaching BSE to relatives and women in the community.

**Contribution:**

It is crucial to provide nurses with the skills and knowledge required to carry out BSE effectively, as well as teach women how to perform it on themselves, to improve breast cancer detection rates in Nigeria.

## Introduction

Breast cancer is the most common cancer and the leading cause of cancer-related death for women worldwide (Azamjah, Soltan-Zadeh & Zayeri 2019; Fitzmaurice et al. 2015; World Health Organization 2022), with the incidence and mortality rates varying by geographical region (DeSantis et al. 2015; Lei et al. 2021; Luo et al. 2022; Torre et al. 2016). It is worth noting that breast cancer mortality rates are highest in regions of Africa and Oceania, which include primarily low- to middle-income countries (LMIC) and low-income countries (LIC), ranging from 17.4 to 20.1 deaths per 100 000 (DeSantis et al. 2015; Lei et al. 2021; Luo et al. 2022; Torre et al. 2016). Although breast cancer mortality rates have decreased over time in most high-income countries (HIC), they remain high and are increasing in many LMIC and LIC (Hashim et al. 2016).

In Africa, the 2020 estimates documented 11 million new cancer cases and 711 429 cancer-related deaths, with females accounting for more than half of both cases (Sharma et al. 2022). It has also been noted that Nigeria ranks as the second-leading country in new cancer cases and mortality on the continent (Sharma et al. 2022). Breast cancer is also the leading cancer among females in Africa (Sharma et al. 2022). Breast cancer incidence has surpassed that of cervical cancer in terms of hospital incidence in Nigeria, and the country has been documented to have the highest breast cancer mortality rate (Azubuike et al. 2018). Breast cancer prevalence in Nigeria has been documented at 116 cases per 100 000 women per year (Adebamowo & Ajayi 2000). In recent studies, although Nigerian women have been found to have heard about breast cancer, the knowledge related to screening methods such as mammograms was lacking (George et al. 2019), and in studies where screening methods were known by the respondents, the utilisation of screening methods such as breast self-examination (BSE) and mammography was minimal (Hanson, Abd El-Kader & Ilesanmi 2019; Ohaeri & Aderigbigbe 2019). Similarly, among diverse categories of women (low, middle and high socio-economic classes), understanding of risk factors and early warning signals of breast cancer was below average (Onwere et al. 2009).

Early detection and prevention of breast cancer are critical for saving lives and enhancing the quality of life (Okobia et al. 2006). Breast cancer diagnosis at an early stage gives women additional treatment options and a better likelihood of long-term survival with better health outcomes (Getu et al. 2018; Li & Shao 2015). As noted by Oluwatosin and Oladepo (2006), breast cancer lends itself to early detection and subsequent treatment if women use early detection measures. Therefore, an effective screening programme is required to lower the incidence of breast cancer deaths. Empowering women and raising awareness among them might go a long way towards improving the breast cancer screening programme. The screening methods for breast cancer include BSE, clinical breast examination (CBE) and mammography (Jemal et al. 2006; Ohaeri & Aderigbigbe 2019).

Breast self-examination is an essential, low-cost and simple tool for detecting breast cancer early (Ohaeri & Aderigbigbe 2019). Early detection improves the prognosis and reduces the associated morbidities and mortalities (Hanson et al. 2019; Ohaeri & Aderigbigbe 2019). It has been found that women who practice frequent BSE are more likely to present clinically with early tumours and have a shorter patient delay in presentation (Oluwatosin & Oladepo 2006). Early breast cancer detection also improves the quality of life and survival (Li & Shao 2015). According to another study by Parvani (2011), women who thoroughly checked their breasts found tiny lumps of breast cancer and their prognosis improved. Parvani (2011) further noted that the respondents who conducted BSE were found to have reported their symptoms to health officials earlier than the others and had an earlier diagnosis and prompt treatment.

However, it is noted that in most developing countries, because of limited resources and poor infrastructure, routine mammography is often not readily available (Jemal et al. 2006; Ohaeri & Aderigbigbe 2019). As the most cost-effective and readily available screening method, BSE is a recommended screening method for the early detection of breast cancer; therefore, it is essential to educate women about BSE as an early detection method for breast cancer (Hanson et al. 2019; Parvani 2011). According to Kösters and Gøtzsche (2003) and Ohaeri and Aderigbigbe (2019), practising BSE helps women identify the composition and structure of their normal breasts. This ensures women’s heightened sensitivity to detect any abnormality at the earliest possible time. Monthly BSE is recommended for women 20 years of age and older to detect new lumps and other breast changes. This contributes to a woman’s heightened awareness of what is normal for her. However, literature has noted that in most cases, even when BSE is done, the practice is not always correct (Ohaeri & Aderigbigbe 2019). Therefore, it is crucial to teach relatives the importance of BSE as a primary tool in screening and early detection of breast cancer (Abo Al-Shiekh, Ibrahim & Alajerami 2021; Olgun & Dizer 2021). As noted by Head et al. (2018), nurses, including student nurses, should be at the forefront of educating the public and engaging in health-related conversations with their own relatives. Furthermore, nursing students who have undergone training on BSE and its benefits are better placed to educate their own relatives in the community; hence, the assumption that if students know the value of BSE they would want to share with those close to them. Breast self-examination alone is believed to be an appropriate and effective method of ensuring early detection of breast cancers; therefore, proper enlightenment and teaching to women, especially relatives of all ages and groups, is critical. It is vital to adequately motivate relatives to regularly carry out BSE to curtail the increasing mortality rate from breast cancer. The practice of BSE can also be utilised to enhance breast cancer awareness among women (Olgun & Dizer 2021). Furthermore, it is important to note that regular BSE is part of the overall health promotion concept (Marmot et al. 2013).

Considering the burden of breast cancer and low levels of breast cancer screening, a change in the behaviour of women is needed to increase the practice of BSE, CBE and mammographic screening (Spiegel, Hill & Warner 2009). As mammography is not readily available in an environment with limited resources like Nigeria, worried by this prevailing situation and with recent data suggesting that health behaviour may be influenced by the level of awareness about breast cancer, it is crucial to find ways of strengthening BSE. Professional nurses and midwives, together with student nurses and midwives, are part of the health care providers and engage with women throughout their reproductive lives and are capable of influencing their health behaviour; hence, they are well placed to fulfil that role (Ossai et al. 2019). Furthermore, Ossai et al. (2019) suggested that women who are advised about BSE and breast awareness by healthcare professionals demonstrate excellent knowledge and confidence and are more likely to practice this procedure routinely than those who became aware of it from other sources. This necessitated the need to assess the practice and teaching of BSE to relatives of students at a college of nursing and midwifery in a north-central state of Nigeria.

## Research methods and design

### Study design

This study applied a cross-sectional, descriptive, quantitative research design to assess nursing students’ practice and teaching of BSE to relatives. Questionnaires were completed by students registered at a College of Nursing and Midwifery in a north-central state of Nigeria. The study is a survey, and the variables of interest lend themselves to the use of a questionnaire for data collection.

### Study setting

This study was carried out from 18 to 22 April 2022, at a college of nursing and midwifery. The selected college was based on proximity; it was the only nursing institution located within the state headquarters out of the three in the state. Since the calculated sample size was 212, 5 days (18 to 22 April 2022) were considered adequate for data collection. Thus, only students who were not within the campus (either hostels or classrooms) at the time of data collection did not participate. Usually, some sets of students are on clinical postings to different accredited hospitals, while others are on study blocks at different times. The college is located at a general hospital in one north-central state of Nigeria and functions as an integral part of the Ministries of Health and Higher Education.

### Population and sampling

The target population for this study were students registered at a college of nursing and midwifery in one north-central state of Nigeria. The college has a total population of 450 eligible female respondents who were students in the study setting.

The sample size was determined using Taro Yamane’s proportion formula: *n* = *N* / 1 + *N* (*e*) 2, where ‘*n*’ = sample size, ‘*N*’ = total population and ‘*e*’ = 0.05.

Thus, the calculated sample size was approximately 212, but 197 nursing students were sighted and consented to participate in the study by completing the administered questionnaires. Therefore, the response rate was 92.9%.

An incidental sampling technique (which is a type of non-probability sampling) was applied to select 197 students in a college of nursing and midwifery in one north-central state of Nigeria. The students selected were those present on campus at the time of data collection, and as previously used in other non-experimental studies, the incidental sampling technique was used in selecting respondents for the study (Anggraini, Rejeki & Puspitaningrum 2021). This technique permits the researcher to utilise those subjects present without going through the randomisation process.

### Data collection tool

A structured questionnaire written in English was designed by the researchers to obtain data from the respondents, with English being the language of instruction and communication in the college and in Nigeria, where the study was carried out. Questionnaire was considered suitable and appropriate as the study was a cross-sectional descriptive quantitative survey. The data collection tool had four sections with 31 questions in all. Section A collected data on respondents’ socio-demographic profile; Section B collected information on respondents’ personal BSE performance; Section C collected data on knowledge about BSE and Section D collected data on teaching BSE to relatives.

The questionnaire was developed by the researchers based on information from the literature. Furthermore, content validity was assured by ensuring that all aspects to be investigated in the study were included in the questionnaire. Once the questionnaire was developed, it was scrutinised by the project supervisor to determine its validity before the questionnaires were administered. The reliability of the data collection tool was established using Cronbach alpha; thus, the reliability coefficients of the research tool section-by-section (B, C and D) were 0.82, 0.85 and 0.88, respectively.

### Data collection

After obtaining ethical approval from the institution ethical committee, respondents were recruited from among the students during lunch hour within the school premises to prevent interruption of class lectures. Each level of student has a class representative. The questionnaires were distributed to each level of class representative, who assisted in administering the questionnaires to his or her respective class members. Information on the purpose of the study was provided at the top of each copy of the questionnaires in the form of written informed consent. Acceptance and completion of the questionnaires were taken as signs of consent by each respondent. Completed questionnaires were retrieved from the respondents by the class representative for delivery to any of the available researchers. Participation was voluntary; anonymity, privacy and all ethical principles were adhered to.

### Statistical analysis

The collected data were entered into Statistical Package for the Social Sciences IBM SPSS Statistics (Version 21). Frequency tables and consideration graphs were used to describe the study variables. Descriptive statistics such as proportional means and inferential statistics, for instance, the chi-square test were used to determine the association between variables. A *p*-value < 0.05 was considered statistically significant.

### Research hypotheses

There is no significant association between knowledge of BSE and practice among student nurses and midwives.

Decision rule: if *p*-value > 0.05, accept the null hypothesis; if *p*-value < 0.05, reject the null hypothesis and accept the alternative.

### Ethical considerations

Ethical approval was obtained from the institution’s ethical committee, where the study was conducted; the approval number being KWSCN/RD/038. During data collection, respondents were given an information document explaining the study purpose, the risks and benefits associated with participation in the study, and the procedures involved. Written informed consent was also obtained from all respondents before including them in the study for data collection. Confidentiality and respondents’ liberty to quit at any study stage were reiterated and maintained. Names and signatures of respondents were not required on the informed consent section, which was at the top of the questionnaire, in order to ensure anonymity. Researchers were available to collect and check the completeness of the retrieved questionnaires daily. Data obtained were stored in well-secured cabinets, and soft copies of the dataset in SPSS software were password protected and made accessible to only the study investigators, with the analyst being the chief investigator.

## Results

Out of the 212 questionnaires administered, 197 were properly completed and entered into computer-based SPSS software for data analysis. Thus, the response rate was set at 92.9%.

### Socio-demographic characteristics of respondents

Table 1 illustrates the socio-demographic characteristics of respondents. The results of the study indicate that 84.8% (*n* = 167) of the respondents were between the age of 18 and 25 years, while the remaining were between the age of 26 and 36 years. Besides, 82.7% (*n* = 163) were in their 3rd year; 85.3% (*n* = 168) were single, with 91.9% (*n* = 181) of them enrolled in the general nursing programme. Furthermore, 14.2% (*n* = 28) of them indicated that they had a positive family history of breast cancer.

**TABLE 1:** Socio-demographic characteristics of respondents (*N* = 197).

Socio-demographic characteristics	Frequency	%
**Age group**		
18–25 years	167	84.8
26–36 years	30	15.2
**Year of study**		
1st year	15	7.6
2nd year	19	9.6
3rd year	163	82.7
**Marital status**		
Single	168	85.3
Married	28	14.2
Divorced	1	0.5
**Religion**		
Christianity	91	46.2
Islam	106	53.8
**Program of study**		
General nursing	181	91.9
Midwifery	16	8.1
**Family history of breast cancer**		
Breast cancer present	28	14.2
Breast cancer absent	169	85.8

### Respondents’ levels of breast self-examination knowledge and practice

**FIGURE 1:**
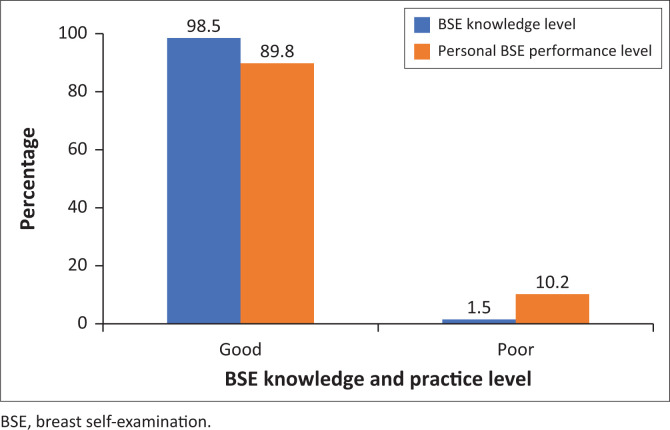
Respondents’ level of breast self-examination knowledge and practice.

The results of this study showed that a total of 98.5% (*n* = 194) of the respondents reported good knowledge of BSE. A slight decrease was noted with regard to BSE performance, with 89.8% (*n* = 177) of the respondents reporting good practice of BSE, while a total of 10.2% (*n* = 20) reported poor practice of BSE. Figure 1 shows respondents’ levels of knowledge and personal BSE practice.

### Respondents’ willingness to teach breast self-examination to relatives

In this study, 73.6% (*n* = 145) of the respondents reported that they had taught BSE to a relative before data collection, while the remaining population had not (Table 2). There were 5.6% (*n* = 11) of the respondents who stated they would not teach BSE to their relatives even if they had the opportunity, claiming the reason they would not teach BSE to a relative even if they had the opportunity was based on lack of training (1.5%; *n* = 3) and lack of familiarity (5.6%; *n* = 10) (Table 3).

**TABLE 2:** Respondents’ willingness to teach breast self-examination to relatives.

Respondents’ willingness to teach BSE to relatives	Yes		No	
	Freq.	%	Freq.	%
Have you taught BSE to a relative before?	145	73.6	52	26.4
Would you teach BSE to a relative if you had the opportunity?	186	94.4	11	5.6

BSE, Breast self-examination; Freq., Frequency.

**TABLE 3:** Respondents’ teaching of breast self-examination to relatives.

Respondents’ teaching of BSE to relatives	Frequency	%
**Focus of BSE teaching to a relative**		
Change in appearance and shape only	14	7.1
Discharge from nipple only	1	0.5
Lump only	13	6.6
All of the above	148	75.1
Not applicable	21	10.7
**Reasons for not teaching BSE to a relative before**		
No time	7	3.6
They did not request for it	29	14.7
They won’t take me serious	16	8.1
Not applicable	145	73.6
**Reasons for unwillingness to teaching BSE to a relative even if there is an opportunity**		
I have not been specially trained on it	3	1.5
Familiarity	10	5.1
Not applicable	184	93.4

BSE, Breast self-examination.

### Respondents’ teaching of breast self-examination to relatives

The focus of the BSE teaching session, as reported by respondents, varied. The result shows that according to the respondents who claimed that they had taught BSE to relatives, 7.1% (*n* = 14) were looking for change in appearance and shape only; 6.6% (*n* = 13) were looking for lumps only and 75.1% (*n* = 148) claimed they were looking for all signs, such as change in appearance, change in shape, discharge from the nipple and palpable lumps (Table 3).

Among the participants in this study, there was no relationship noted between their knowledge of BSE and their practice (*X*^2^ = 16.509, df = 1, *p* = 0.28 > 05).

## Discussion

Breast cancer is an epidemic affecting millions of women, with an exceptionally high mortality rate in LMIC like Nigeria. There is, therefore, an imminent need to promote the use of primary preventive methods such as BSE to aid in the prevention, early detection and prompt treatment of breast cancer. Results from this study indicate that the most significant proportion of the respondents were aged between 18 and 25 years; the minimum age allowed for students to be admitted into higher education in Nigeria is 16 years. Therefore, the age range is appropriate for the category of students who happened to be respondents in this study. Besides, more than 80% were in their 3rd year; the majority of the students in 1st and 2nd had been deployed to hospitals for clinical experiences. Similarly, over 85% were single, because a higher percentage of them were still young and, as such, were expected to devote quality time to their educational careers with little or no distractions. Also, most of them had been working as nurses for 3 years, with a number of them stating a positive family history of breast cancer.

Results from the study showed that most respondents reported good practices in BSE, while a few reported poor practices. In disagreement with the findings from this study are the findings from the study by Nde et al. (2015) on undergraduate students at the University of Buea, where nearly three-quarter of the respondents had previously heard of BSE, yet only 9% knew how to perform it, and only 3% performed it regularly. The findings of Gilani et al. (2010) also found poor (28.3%) practices regarding BSE among the outpatients’ department in Pakistan, which is in discord with the findings from this study. The high self-reported performance of BSE in this study may be a result of the majority being in their 3rd year, almost becoming professional practising midwives, and having acquired the knowledge and skills more recently. This is further supported by findings from the study by Alsaif (2004) in Riyadh, where about 66% of the nursing students reported regular performance of BSE, highlighting regular BSE practice among student nurses and indicating the opportunities to apply what is learned in their own lives.

Further, results from this study also showed that most of the respondents reported that they had good knowledge of BSE. In the study of Al Junaibi and Khan (2011), who researched knowledge and awareness of breast cancer among female university students in Oman, over 70% knew that BSE should be performed monthly. This corresponds with the current study’s findings. This study also agrees with the study of Florence et al. (2016) among university students in Benin City, Nigeria, where the majority of the respondents (93.5%) had adequate knowledge about BSE. However, unlike this study, limited knowledge about breast cancer and BSE among female medical students in Saudi Arabia was identified by Nemenqani et al. (2014). It is important to establish BSE knowledge among nursing students and other healthcare providers, as lack of knowledge was cited as the main reason for the poor practice of BSE (Nde et al. 2015). Therefore, in a collective effort to ensure early detection of breast cancer, there is a need to encourage and disseminate adequate information about BSE continually among all healthcare providers, who are tasked with the responsibility to disseminate such information not only to their clients and patients but also to their relatives. This emphasis on ensuring that BSE information is shared with women of all categories has been recommended by numerous studies, as such endeavour has great potential in equipping and supporting women to conduct regular BSE, hence ensuring early detection and better health outcomes for women (Hanson et al. 2019; Li & Shao 2015; Ohaeri & Aderigbigbe 2019).

Moreso, results from the study show that most respondents had previously taught BSE to a relative while the remaining population had not. Reasons that discourage respondents from teaching their relatives include a lack of confidence and a lack of specialist training. The study of Karayurt, Özmen and Çetinkaya (2008) and Ferlay et al. (2004) identified several reasons like fear of possible discovery of a lump, lack of time, lack of self-confidence in their ability to perform the technique correctly and embarrassment associated with manipulation of the breast as reasons for not practising and teaching BSE, which supports findings from this study. Among the respondents who reported that they had taught BSE to relatives, a small percentage of them stated they asked their relatives to look out for changes in appearance and shape only, while some of the respondents stated they asked their relatives to look out for lumps only. However, the greatest proportion of them stated they asked their relatives to look out for all signs, ranging from changes in appearance to discharge from the nipple and signs of lumps. This indicates a robust detection of symptoms of breast cancer as highlighted by the American Cancer Society (2017), including breast pain, swelling, thickening, presence of a painless lump or redness of breast skin, nipple retraction and nipple discharge. These symptoms can be easily detected with BSE when done regularly. Hence, the importance of BSE cannot be overemphasised. It is important to ensure that the information given out about BSE is complete, highlighting step-by-step procedures that will increase the benefits of conducting BSE (Hanson et al. 2019; Ohaeri & Aderigbigbe 2019). Furthermore, nursing students need to be empowered to ensure they provide BSE information that is appropriate to their audience, whether relatives or clients; this will ensure targeted education activities as well as useful health information geared towards the screening of breast cancer (George et al. 2019; Lei et al. 2021). This is crucial as members of community need the continuous support from healthcare providers who are well placed to strengthen health promotion in the community and student nurses are well placed to act even within their own families and enhance the practices of BSE.

The significant association noted between knowledge of BSE and the practice of BSE among student nurses and midwives indicates the need for student nurses, who will be future nurses, to have adequate knowledge of BSE. This association between knowledge and practice of BSE is supported by previous studies in different countries and serves as a motivation to ensure BSE knowledge is increased in all spheres of women’s lives (George et al. 2019; Hanson et al. 2019).

## Conclusion

Breast self-examination is an essential procedure to ensure early detection of breast cancer, prevent its delayed diagnosis and improve health outcomes, as early diagnosis is associated with a better prognosis. However, although the self-reported BSE level of knowledge and practice was high, it has been shown that some student nurses are not even interested in teaching BSE to their families and loved ones as they consider familiarity and lack of confidence to be considerable barriers to its implementation. This study sheds additional information on BSE and its education for health professionals, particularly nurses. Teaching BSE to one’s relatives could be a safe place for continuous practice, as this is a required type of education for clients in the healthcare institution where the student nurses will be working after graduation.

## Recommendations

Based on the findings of this study, the following recommendations are made:

1. Student nurses need to be encouraged and empowered to implement what they learn in their day-to-day lives; hence, increasing awareness of the need for health professionals to teach BSE to relatives should be reinforced, as it would strengthen their practice when in the clinical setting.
2. Health education on BSE and other breast cancer screening strategies should be extended to secondary schools and higher institutions of learning so that all women can have an understanding of the importance of regular and correct BSE from a young age.
3. Workshops and seminars should be organised to teach nurses the importance of teaching BSE to relatives and the larger world of women. Regular simulation sessions on breast examination would assist the student nurses in gaining confidence to teach BSE to their relatives and clients.
4. Healthcare providers should equip themselves with updated skills on BSE performance and teaching models.

## Limitations

The use of a structured questionnaire developed by the researchers, which relied on face, and content validities, with limited opportunity to establish the reliability of the instrument, creates a limitation to the study. Furthermore, the use of non-probability sampling also removes the possibility of generalising the findings. There was over-recruitment at the 3-year level, which does not allow generalisation of the findings to the whole institution and to the population of nursing students in general.

The questionnaire collected information on self-reported knowledge and performance with regard to BSE. The strength of the results relies on the respondents answering truthfully to the questions and making the correct judgement on their level of knowledge and performance. As data collection was anonymous and the researchers worked with the year-level representative, that would have reduced the risk of the respondents giving what would be seen as acceptable answers, as there were no power relations exhibited during the process of data collection.
